# The Association of Sensory Impairment With Incident Disability-Related Cessation of Employment in Older Adults

**DOI:** 10.1093/geroni/igaa057.697

**Published:** 2020-12-16

**Authors:** Ahmed Shakarchi, Emmanuel Garcia Morales, Nicholas Reed, Bonnielin Swenor

**Affiliations:** 1 Johns Hopkins Wilmer Eye Institute, Baltimore, Maryland, United States; 2 Johns Hopkins Bloomberg School of Public Health, Baltimore, Maryland, United States; 3 Johns Hopkins University, Baltimore, Maryland, United States

## Abstract

Sensory impairment (SI) is common among older adults, and it is an increasingly important public health challenge as the population ages. We evaluated the association between SI and incident disability-related cessation of employment in older adults using the population-based Health and Retirement Study. Participants employed in 2006 completed biennial interviews until self-reported incident disability-related cessation of employment. Participants were censored at loss to follow-up, retirement, or 2018. Participants rated their vision and hearing, using eyeglasses or hearing aids if applicable, on a Likert scale (poor, fair, good, very good, excellent). SI was defined as poor or fair ability, and SI was categorized as neither SI (NSI), vision impairment alone (VI), hearing impairment alone (HI), and dual SI (DSI). Cox proportional hazard regression assessed the association between SI and incident disability-related cessation of employment, adjusting for demographic and health covariates. Overall, 4726 participants were included: 421 (8.9%) were with VI, 487 (10.3) with HI, and 203 (4.3%) with DSI. Mean age was 61.0 ± 6.8 years, 2488 (52.6%) were women, and 918 (19.4) were non-White. In the fully adjusted model, incident disability-related cessation of employment over the 12-year follow-up period was higher in VI (Hazard Ratio (HR)=1.30, 95% confidence interval (CI)=0.92, 1.85), HI (HR=1.60, CI=1.16, 2.22), and DSI (HR=2.02, CI=1.38, 2.96). These findings indicate that employed older adults with SI are at increased risk of incident disability-related cessation of employment, and that older adults with DSI are particularly vulnerable. Addressing SI in older adults may lengthen their contribution to the workforce.

